# Effectiveness of a theory-based back care intervention on spine-related behavior among pupils: a school-based randomised controlled trial (T-Bak study)

**DOI:** 10.1186/s12889-020-08566-z

**Published:** 2020-05-29

**Authors:** Zahra Akbari-Chehrehbargh, Sedigheh Sadat Tavafian, Ali Montazeri

**Affiliations:** 1 Department of Health Education, Faculty of Medical Sciences, Tarbiat Modares University, Tehran, Iran; 2 Health Metrics Research Center, Iranian Institutes for Health Sciences Research, ACECR, Tehran, Iran; 3 Faculty of Humanity Sciences, University of Science &Culture, ACECR, Tehran, Iran

**Keywords:** Social cognitive theory, Backache, Educational programme, Behavior, Schoolchildren

## Abstract

**Background:**

Children’s health and welfare have a special place in research and policy in many countries. One of the most important concerns is the increasing rate of backache in children due to many of behavioral risk factors. The aim of this study was to evaluate the effectiveness of an educational program on promoting back-related behavior as well as knowledge, skills, beliefs, and self-efficacy among fifth grade girls.

**Methods:**

The theory-based back care (T-Bak) study was a school-based randomised controlled trial (RCT) that assessed the effectiveness of developing a back care training program based on the social cognitive theory (SCT). A total of 104 schoolchildren aged 11 ± 1.0 years were assigned to intervention (*n* = 52) and control (*n* = 52) groups. The intervention group received six sessions training on proper lifting and carrying techniques, having proper posture during daily activities, and correct backpack wearing techniques with a 1-week interval while the control group received nothing. Then, the two groups were assessed for knowledge, skills, self-efficacy, beliefs, and behavior at four points in time: baseline, immediate, three and six-months post-intervention. The changes of the outcomes investigated using univariate repeated measures analysis of variance. Partial eta squared measure (η_p_^2^) was used to calculate effect sizes.

**Results:**

A positive change was found for the intervention group back-related behavior from baseline to immediate post-intervention and follow-ups (*F* = 78.865, *p* < 0.001, η_p_^2^ = 0.22). Overall there were 36.4% improvement for knowledge (η_p_^2^ = 0.21), 53.2% for the skills (η_p_^2^ = 0.25), 19.5% for the self-efficacy (η_p_^2^ = 0.11), and 25.6% for the beliefs (η_p_^2^ = 0.14) scores from baseline to 6 months’ follow-up assessments among the intervention group (*p* < 0.001). The results also showed a significant interaction effect between group and time.

**Conclusion:**

The T-Bak intervention was effective in improving back-related behavior in pupils. It is now available and could be evaluated further in back-care related studies.

**Trial registration:**

Current Controlled Trials IRCT20180528039885N1, 30th Oct 2018, ‘Prospectively registered’. https://www.irct.ir/trial/31534

## Background

Back pain is a major public health problem [1–6] on the rise among all ages including adolescents and in particular pupils [1, 3, 7–9]. Lifetime prevalence of this condition in this age group also varies from 13 to 51% [4]. It is well known that back pain in younger generation might be due to genetics and trunk asymmetry in children and adolescents especially girls as well as several behavioral risk factors including adoption of improper postures during sitting, standing, and lifting heavy objects, carrying heavy backpacks incorrectly, carrying school bags on one side of the body, and sedentary lifestyle [1, 3, 10].

Therefore, back pain educational programs have been developed and evaluated for elementary school children [7, 11–21]. For example, Cardon et al. used a school-based educational program on back care principles among Belgian pupils in which children were taught through guided discovery and active hands-on methods such as games and dramatic plays [18]. In a recent study by Dullien et al., an evaluation by a teacher through a multi-part school-based back care educational program among German schoolchildren revealed that self-reported back pain had not decreased during one-month follow-up. Statistically significant improvement had been found in back care knowledge and some behaviors from pre- to post-test stage; however, there had been no statistically significant difference in sitting postures and using heavy school bags [3]. Santos et al. [21] and Dolphens et al. [13] also investigated the effects of a spine care educational program in Brazilian and Belgian schoolchildren; respectively. As reported by Santos et al., there had been no statistically significant difference between post-test and follow-up assessments of knowledge and postures during daily living activities [21]. Similarly, Dolphens et al. reported that back care educational program was effective in improving cognitive determinants of back care, but no changes had been observed in actual behavior or self-efficacy [13]. The results established by Franz et al. also suggested that knowledge about back pain risk factors in childhood might lead to early prevention. They even argued that changes in actual back care-related behavior among children was very difficult [15] since such changes would be more likely to occur when there were subsequent changes in cognitive determinants of behavior [22].

However, a key limitation of these investigations was the fact that they had not benefited a theory for their interventions. In general, it is possible to claim that spine-related behavior educational program in elementary schools has been scarcely examined from a theoretical point of view. In fact, most studies [7, 11–21] had not taken account of potential change strategies for back care behavior and its main determinants. It was decided that it would be more possible to achieve the desired changes in back care behavior during daily activities if there was a theory behind the design of such interventions.

Among behavior change theories, the Social Cognitive Theory (SCT) seems very relevant to developing interventions for back care interventions for schoolchildren [22]. According to this theory, three main psychological determinants of behavior are i. behavioral capability (knowledge and skills to perform a given behavior), ii. self-efficacy (SE), and iii. Outcome expectations (beliefs) [23, 24]. In fact, the theory contains those constructs that engage individuals in a given behavior [23, 24]. Since schoolchildren are also very prone to adopt new behaviors, it gives the impression that the SCT is a good platform for developing educational programs [22, 25]. There are also promising results as the SCT has been used for other topics such as nutrition and physical activity in this age group [22]. Since the main constructs of the SCT are an important set of changeable factors assumed to combine in different ways to determine health-related behavior and distinguish between those performing and not performing behaviors [23, 24], effective interventions based on the proposed constructs can be developed for back care-related interventions. Thus, it was hypothesized that offering an educational program on changes in back care-related behavior based on the SCT to schoolchildren might be effective. In particular, this study aimed to propose a new approach (using effective techniques and change strategies) for back care education and to explore the effect of a theory-based back care (T-Bak) educational program on back care-related behavior as well as knowledge, skills, beliefs, and self-efficacy among 5th-grade girls enrolled in public elementary schools in the city of Tehran, Iran.

### Hypotheses

The hypotheses were that the intervention group improved in their back-related behavior, knowledge, skills, beliefs, and self-efficacy compared to the control group.

## Methods

### Trial design

This was a school-based cluster randomised controlled trial (RCT) that was carried out in Tehran, Iran. Tehran has 22 districts. The study was carried out in district 22 (northwest) where a population with a variety of socio-economic background are scattered across the district. The study was conducted in 2018–2019 academic year. The intervention implemented in classrooms.

### Participants

The study participants were female school children. Pupils were eligible to participate in the study if they were 5th grade elementary schoolchildren, aged 11 ± 1.0 years agreed for participation by their school principal and parents; accepted to participate in the study voluntarily; and were able to attend training sessions. Exclusion criteria included: received back care educational program previously; unwilling to participate, any self-reported back pain history and unhealthy spine.

In all 104 pupils entered into the study (52 pupils allocated to the intervention group and 52 to the control group). At immediate post-intervention data collection, 96% (*n* = 100), at 3-months follow-up, 96% (*n* = 100), and at 6-months follow-up, 95% (*n* = 99) of the sample were retained respectively. Three pupils declined to participate and two were absent. The characteristics of participants are shown in Table 1. None of the demographic parameters showed significant differences between two groups at baseline.

**Table 1:** Demographic characteristics data of pupils at baseline measurement

	Control (***n*** = 52)	Intervention (***n*** = 52)	***P*** value*
	No. (%)	No. (%)	
**Father’s job**			0.98
Employed	47 (90.4)	45 (86.5)	
Unemployed	1 (1.9)	4 (7.7)	
Retired	4 (7.7)	3 (5.8)	
**Mother’s job**			0.33
Employed	13 (25.0)	16 (30.8)	
Housewife	39 (75.0)	36 (69.2)	
**Father’s level of education**			0.80
Illiterate/primary	1 (1.9)	1 (1.9)	
Secondary	38 (73.1)	35 (67.3)	
Higher	13 (25.0)	16 (30.8)	
**Mother’s level of education**			0.69
Illiterate/primary	4 (7.7)	2 (3.8)	
Secondary	30 (57.7)	32 (61.5)	
Higher	18 (34.6)	18 (34.6)	
**Kind of habitation**			0.54
Rented	18 (34.6)	21 (40.4)	
Own	34 (65.4)	31 (59.6)	
**Birth rank**			0.58
First child	25 (48.1)	30 (57.7)	
Second child	20 (38.5)	17 (32.7)	
Ohers	7 (13.5)	5 (9.6)	
**Number of family members**			0.43
3 people	10 (19.2)	7 (13.5)	
> 3 people	42 (80.8)	45 (86.5)	
**Transmit tool**			0.19
Walking	13 (25.0)	7 (13.5)	
Public transportation	2 (3.8)	2 (3.8)	
Own car	15 (28.8)	25 (48.1)	
School service	22 (42.3)	18 (34.6)	

* χ^2^ test, significant at < 0.05

### The T-BAK intervention

**Fig. 1:**
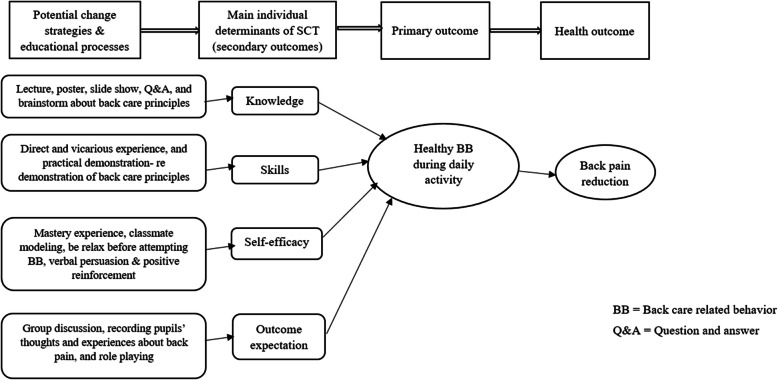
Conceptual framework of the T-Bak study

The study intervention consisted of six sessions, each one lasting for 1 h with a one-week interval, performed by a trained physical education instructor and a health educator. The program was designed based on the main constructs of the SCT and implemented in one class at a time (maximum of 26 pupils). In order to modify the proposed psychological factors in pupils, effective techniques and change strategies could be also employed [23, 24] (Fig. 1). It should be noted that the educational content of the intervention was developed using previous studies [12, 13, 17, 18]. The participants in the control group also received the T-Bak educational program 6 months after the study was completed.

**Fig. 2:**
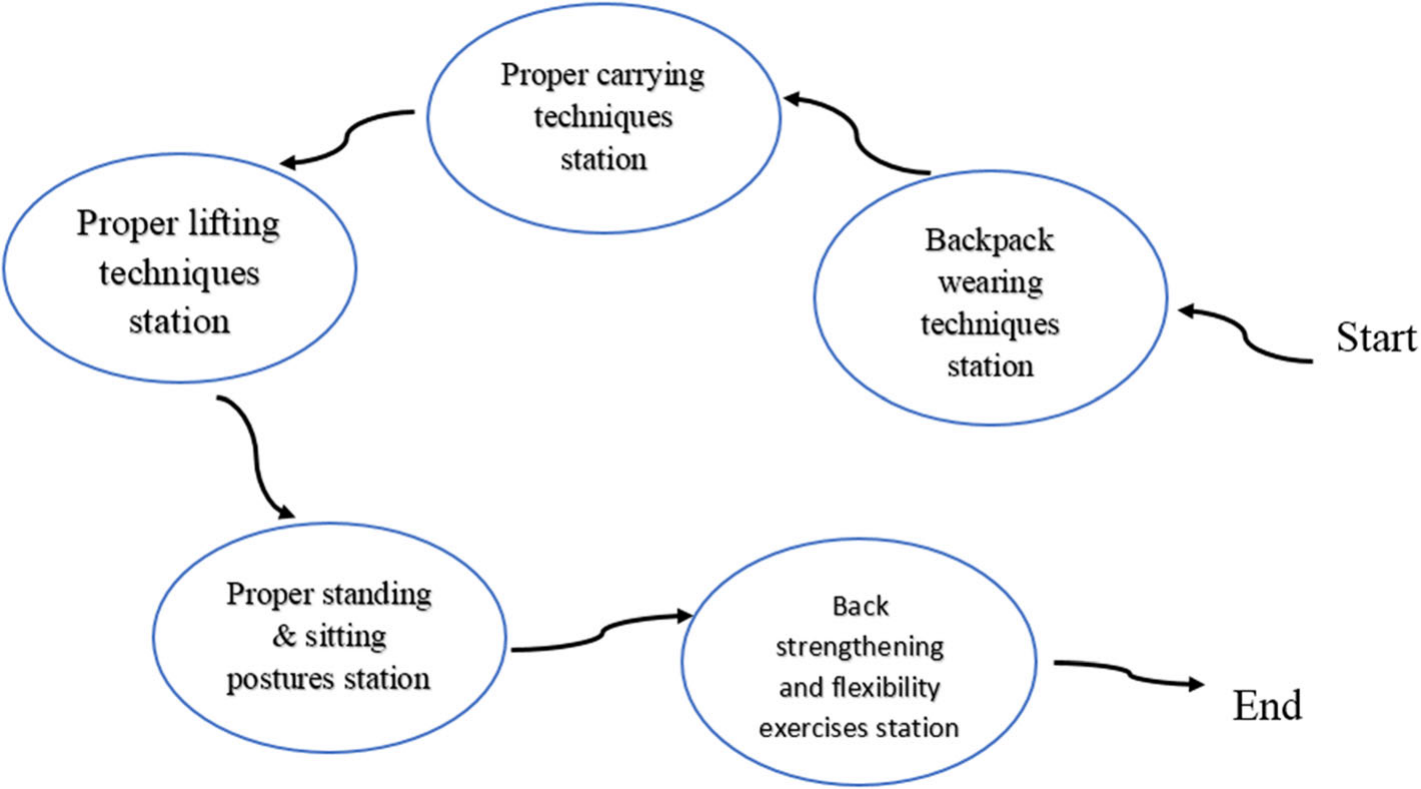
Practice stations for skills-related tasks and self-efficacy improvement

The intervention included four components of beliefs, knowledge, skills, and self-efficacy (Table 2) as explained in the following sections:

- Beliefs: It consisted of one session using group discussion, recording pupils’ thoughts and experiences about back pain, and role-plays when having back pain during sitting, swimming, running, practicing physical activities, cycling, and lifting heavy objects as dangerous tasks. The potential change strategy was positive outcomes of healthy back care-related behavior or introduction of a person with chronic back pain. Pupils also learned how to adopt a healthy back care behavior and principals benefiting them.
- Knowledge: It was comprised of one session including a review of the spine anatomy and focus on the three natural curves in the spine as well as the importance of maintaining an ‘S’ curve vs. a ‘C’ curve during daily activities. In addition, the required back care knowledge was provided through lectures, slide demonstrations, and posters.
- Skills: It involved two sessions including mastery learning through back care essential skills training, using direct and vicarious experiences and practical demonstrations, along with re-demonstration methods. Skills training activities were also designed to increase pupils’ abilities to accomplish back care-related behaviors.
- Self-efficacy: It contained two sessions using mastering practices, observing others’ performance, receiving suggestions from others, and confronting emotions arising from thoughts of change. Moreover, there were attempts to improve pupils’ beliefs about their abilities to perform back care-related behavior. For this purpose, the back-related behavior was divided into smaller sections (Fig. 2) and the above-mentioned change strategies were employed. As well, self-efficacy provided practical experiences to fulfill back care-related behavior successfully.

**Table 2:** Description of intervention content and change strategies

	Content	Change strategies
Session 1. (Improvement of beliefs)	Benefits of healthy back behavior and back pain prevention. When having a backache, sitting, swimming, running, participating in physical education, cycling and lifting heavy objects are dangerous.	- Group discussion
		- Role playing
		- Recording pupils’ thoughts and experiences about back pain
Session 2. (Improvement of knowledge)	A review of the anatomy of the spine focused on the three natural curves in the spine and the importance of maintaining an ‘S’ curve versus a ‘C’ curve during daily activity. Instruction of proper posture during sitting, standing, lifting a load, carrying a load, transferring a load; packing a backpack and correctly carrying a backpack.	- Lecture
		- Brainstorm, Q & A
		- Slides show
		- Posters
		- Pamphlets
Session 3,4. (Improvement of skills)	Mastery learning and practical demonstration of the back care essential skills included:	- Direct experience
		- Vicarious experience
	1- Backpack wearing techniques:	- Demonstration, re- demonstration
	• Using 2 straps.	
	• Firming both straps to keep the pack above the waist.	
	• Balancing the load so the heaviest books are closer to the back.	
	• Not carrying more than 10% of the body weight.	
	2- Maintain a neutral spine:	
	• Keeping the ‘S’ curve.	
	• Avoiding the ‘C’ curve for good posture.	
	3- Lifting techniques:	
	• Keeping feet apart.	
	• Bending the knees and not waist.	
	• Keeping the load close to the body.	
	• Keeping the back straight.	
	• Pushing up with the legs.	
	4- Carrying techniques:	
	• Keeping the load close.	
	• Bending the knees to set the load down.	
	5- Balancing the load:	
	• Using 2 smaller bags instead of 1 large bag.	
	• Carrying items in both hands.	
	6- Picking up a load from a Table:	
	• Pivoting or moving the feet, not twisting.	
	• Keeping the load close.	
	7- Proper sitting posture:	
	• Sitting up straight. Avoiding slouching forward.	
	• Keeping both feet on the floor.	
	• Rolling side to side to feel the ‘sit bones’.	
	8- Proper standing posture:	
	• Standing up straight like a stack of bricks.	
	• Lining up the ear – shoulder – hip – knee – ankle.	
	• Pulling in the belly button to tuck the hips in correctly.	
	• Rolling the shoulders back.	
Session 5,6. (Improvement of self-efficacy)	1- Achieving back strengthening and flexibility exercises.	- Mastery experience
		- Goal setting
	2- Attaining a natural curvature of the spine: • Keeping the ‘S’ curve. • Avoiding the ‘C’ curve for good posture.	- Social modeling
		- Improving physical and emotional states
	3- Minimal loading of the book bag.	- Verbal persuasion
	4- Paying attention to ergonomically postures during sitting, standing, lifting a load, carrying a load, transferring a load, and carrying a backpack.

In order to practice skills-related tasks and to improve self-efficacy, a stationary method was invented and used during a physical education class (Fig. 2). As such, first, five stations were defined including backpack wearing techniques station (station 1 - entry station), carrying techniques station (station 2), lifting techniques station (station 3), proper sitting and standing postures station (station 4), and back strengthening and flexibility exercises station (station 5 - last station). There were also 5 or 6 pupils at each station at one time and they were taught and engaged in the relevant tasks in a sequence. Moreover, the physical education instructors and health educators supervised the stations and subsequently taught the correct tasks as appropriate as possible. The pupils also practiced at the stations until they could perform properly. For all the stations, behavior change strategies had been already predefined.

A major strength of the T-Bak educational program was the use of the SCT depicting the specific activities of an intervention plan designed for the participants. These activities were hypothesized to lead to changes in pupils’ cognitions. It was correspondingly demonstrated how intervention strategies could affect determinants of back care-related behavior. One other strength of the T-Bak educational program was utilizing practice stations for skills-related tasks and self-efficacy improvement that could provide more opportunities for instruction as an innovative method in school-based spinal health interventions.

### Outcomes and measures

In this study, the most feasible outcome measures (i.e. primary and secondary outcomes) were assessed and the long-term impact of the T-Bak intervention on back pain reduction (namely, health outcome) (Fig. 1) was not investigated. The primary outcome was improved back care-related behavior and the secondary outcomes were enhancement in beliefs, back care knowledge and skills, and self-efficacy. Furthermore, the outcomes were assessed using validated instruments [12, 13, 17, 18].

Back care-related behavior: It was tested through six items regarding book bag weight check, use of two straps, everyday exercise, postures while putting on shoes, and postural behavior during lifting and carrying objects. These questions were rated on a five-point Likert-type scale (from never to ever) giving a total score ranging from 6 to 30 in which a higher score indicated a desirable behavior [13].

Beliefs: They were measured through six items about the time experiencing back pain during sitting, swimming, running, practicing physical activities, cycling, and lifting heavy objects as dangerous tasks. The items were also rated on a five-point Likert-type scale with a total score between 6 and 30 [12, 13].

Knowledge: It was assessed through a multiple-choice quiz including 10 items about general and specific back care knowledge based on the content of back promotion program with a total score ranging from 0 to 10 (higher scores denoted higher knowledge) [17].

Skills: A checklist developed by Cardon et al. was used to evaluate back care skills. The skills were accordingly assessed through seven tasks and the pupils could obtain a range of points (from 0 to 46) where higher points suggested more appropriate skills. For each score, specific criteria were also defined. The tasks included (1) sitting at a table, (2) lifting a 3-kg book box from the floor, (3) carrying the book box for a distance of 3 m, (4) putting the book box down on a table, (5) picking up an object from the floor, (6) moving a 3-kg book box from one table to another one, and (7) loading and wearing a backpack [17, 18].

Self-efficacy: It was evaluated by four items asking pupils to indicate how they do perceive healthy back behavior (i.e. daily exercise, accomplishing a natural curvature of the spine, minimal loading of a book bag, and paying attention to ergonomic postures), easy or difficult? The items were also rated on a four-point Likert-type scale (very difficult to very easy) with a total score ranging from 4 and 16 where the higher scores implied higher self-efficacy [12, 13]. Cultural adaptation and psychometric testing were consequently performed.

Outcomes in both groups were assessed at four points in time, namely, at baseline (1 week before intervention), immediate post-intervention (1 week after intervention), and three and 6 months after intervention at school supervised by class teacher. In all instances, a self-reported questionnaire was distributed among the participants, a skills assessment checklist was completed by two independent trained evaluators blinded to the study, and pupils’ data were collected via a short demographic characteristics questionnaire regarding information about parents’ occupation and levels of education, housing, birth order, number of family members, means of transportation (how they were taken to school), as well as two items about presence of LBP during last week (Yes, No) and receiving back care educational program previously (Yes, No).

### Sample size

Sample size estimation was based on previous study on back care education [14]. Based on this study (pre SD = б_1_ = 4.82 & post SD = б_2_ = 4.66) and expecting at least 2-unit difference in mean score of pre and post back care related behavior in intervention group (μ_1_-μ_2_); the following formula was used to estimate sample size.

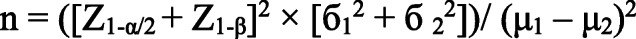



As such a study with 46 participants per group would have 80% power (β = 0.2) at 5% significance level (α = 0.05). However, allowing for a 10% dropout a sample of 52 pupils for each group was thought (in all 104).

### Randomisation

First a list of schools in the district was provided. In all there were eight female public elementary schools. Then, two schools (out of 8) were randomly selected. The schools were numbered as 1 and 2, and numbers were placed in the bowl. An administrative person not connected to the study was blindfolded and asked to pull out one of the numbers from the bowl. The school number 1 was assigned to the interventional group and the second school was assigned to the control group using random numbers. As the final step, since in a school environment, individual randomisation was not possible, the classes of fifth grade (in all 4 classes) in each school were numbered as 1 to 4, and numbers were placed in the bowl. Then two numbers were drawn out of the bowl in a random manner and two classes (out of 4 classes) in each school enrolled the study.

**Fig. 3:**
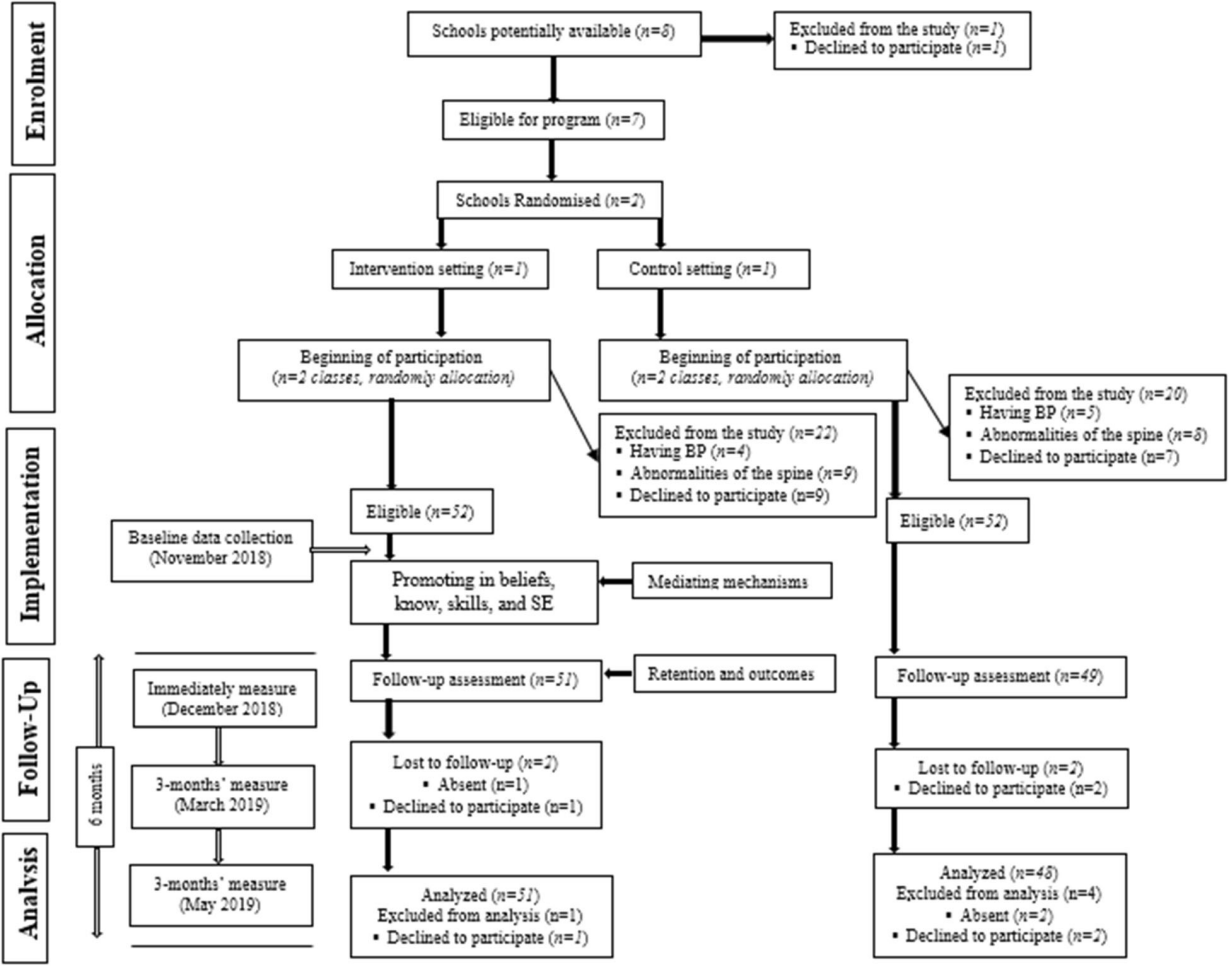
The T-Bak intervention participation flow diagram

The main investigator (ZAC) monitored the randomization procedure. The T-Bak study was performed and reported in accordance with the CONSORT guidelines. The study flowchart is also presented in Fig. 3.

### Statistical analysis

Data analysis was performed using SPSS 24.0 (IBM SPSS Version 24.0. Ink, NY: IBM Corp). Matching between both groups was considered for baseline characteristics and potential confounding variables, such as socioeconomic status, and age. Descriptive statistics were used to explore the data. The Chi-squared analysis was used for comparing categorical variables (demographic characteristics) data. For quantitative data, independent samples t- test was used in order to compare baseline group differences. Repeated measures univariate analysis of variance was performed with ‘time’ as within-subjects factor (at baseline, immediate, 3-months and 6-months follow-ups), and ‘group’ as between-subjects factor (intervention vs. control group). For pairwise comparisons a Tukey’s HSD post hoc test was used to find out which groups differed from each other. Partial eta squared measure (η_p_^2^) was used to calculate effect sizes for all the statistically significant differences. Partial eta squared gives us an idea of how different our samples are. In other words, it tells us about the magnitude of the effect. Usually the following cut-offs are used to interpret partial eta squared: (i) 0.01 to < 0.06 as small effects, (ii) 0.06 to < 0.014 as medium effects, and (iii) 0.14 or more as large effects. The level of significance was set at *p* < 0.05.

## Results

Table 3 shows the mean scores, interaction and main effects of ‘group’ and ‘time’ on outcome variables for the intervention and control groups at baseline, immediate post-intervention, 3 and 6 months’ follow-up. In Table 4, we reported the Turkey’s HSD post-hoc test for all variables.

**Table 3:** Means (SD), interaction and main effects of group and time on outcome variables at baseline, immediate, 3 and 6 months’ follow-ups between intervention and control groups in the T-Bak study

Variable (score range)	Score (mean ± SD)								Interaction effect (F): Time×Group	Time (F)	Group (F)
	Baseline		Immediate		3-months follow-up		6-months follow-up				
	Intervention (*n* = 52)	Control (*n* = 52)	Intervention (*n* = 51)	Control (*n* = 49)	Intervention (*n* = 50)	Control (*n* = 50)	Intervention (*n* = 51)	Control (*n* = 48)			
Behavior (6–30)	17.26 ± 4.97	18.30 ± 5.00	26.35 ± 3.61	17.20 ± 5.59	26.68 ± 3.58	18.30 ± 4.84	26.84 ± 2.95	18.08 ± 5.13	29.266*	24.693*	192.985*
***P*-value**	0.36		**< 0.001**		**< 0.001**		**< 0.001**				
Knowledge (0–10)	4.16 ± 1.53	4.30 ± 1.46	7.45 ± 1.83	4.16 ± 1.61	7.82 ± 1.8	4.16 ± 1.53	7.80 ± 1.84	4.35 ± 1.65	29.395*	27.278*	235.725*
***P*-value**	0.65		**< 0.001**		**< 0.001**		**< 0.001**				
Skills (0–46)	13.26 ± 9.37	13.70 ± 10.18	38.75 ± 10.30	13.53 ± 10.18	38.20 ± 11.83	12.48 ± 9.29	37.73 ± 11.63	12.79 ± 9.68	38.556*	34.229*	330.483*
***P*-value**	0.95		**< 0.001**		**< 0.001**		**< 0.001**				
Self-efficacy (4–16)	10.66 ± 2.86	10.2 ± 2.97	14.22 ± 2.17	10.80 ± 2.73	13.68 ± 2.30	9.90 ± 3.11	13.7 ± 1.88	10.44 ± 3.05	9.985*	11.512*	104.833*
***P*-value**	0.66		**< 0.001**		**< 0.001**		**< 0.001**				
Beliefs (6–30)	19.16 ± 4.19	18.08 ± 4.83	26.31 ± 4.39	18.18 ± 4.42	26.32 ± 5.09	18.06 ± 5.02	26.84 ± 4.40	18.67 ± 4.62	14.692*	16.879*	190.811*
***P*-value**	0.24		**< 0.001**		**< 0.001**		**< 0.001**				

*SD* Standard deviation

*P < 0.001

**Table 4:** Tukey multiple comparisons for all variables in intervention group

Variable (score range)	(I) Time	(J) Time	Mean Difference (I – J)	***P***-value	95% Confidence Interval for Difference	
					Lower Bound	Upper Bound
Behavior (6–30)	Baseline	Immediate	−3.999^a^	**< 0.001**	− 5.260	− 2.737
		3-months follow-up	−4.710^a^	**< 0.001**	− 5.972	− 3.448
		6-months follow-up	−4.683^a^	**< 0.001**	−5.948	−3.418
	Immediate	3-months follow-up	−0.711	0.61	−1.973	0.550
		6-months follow-up	−0.685	0.33	−1.950	0.580
	3-months follow-up	6-months follow-up	0.027	0.94	−1.238	1.292
Knowledge (0–10)	Baseline	Immediate	−1.577^a^	**< 0.001**	−2.041	−1.113
		3-months follow-up	−1.760^a^	**< 0.001**	− 2.224	− 1.296
		6-months follow-up	−1.849^a^	**< 0.001**	−2.314	−1.384
	Immediate	3-months follow-up	−0.183	0.29	−0.647	0.281
		6-months follow-up	−0.272	0.41	−0.737	0.193
	3-months follow-up	6-months follow-up	−0.089	0.75	−0.554	0.376
Skills (0–46)	Baseline	Immediate	−12.658^a^	**< 0.001**	−15.538	−9.778
		3-months follow-up	−11.860^a^	**< 0.001**	−14.740	−8.980
		6-months follow-up	−11.779^a^	**< 0.001**	− 14.666	−8.891
	Immediate	3-months follow-up	0.798	0.86	−2.082	3.678
		6-months follow-up	0.879	0.70	−2.009	3.767
	3-months follow-up	6-months follow-up	0.081	0.85	−2.806	2.969
Self-efficacy (4–16)	Baseline	Immediate	−2.036^a^	**< 0.001**	−2.777	−1.294
		3-months follow-up	−1.320^a^	**0.001**	−2.062	−0.578
		6-months follow-up	−1.641^a^	**< 0.001**	−2.385	− 0.897
	Immediate	3-months follow-up	0.716	0.15	−0.026	1.457
		6-months follow-up	0.395	0.19	−.349	1.139
	3-months follow-up	6-months follow-up	−0.321	0.78	−1.065	0.423
Beliefs (6–30)	Baseline	Immediate	−3.629^a^	**< 0.001**	−4.918	−2.340
		3-months follow-up	−3.570^a^	**< 0.001**	−4.859	−2.281
		6-months follow-up	−4.135^a^	**< 0.001**	−5.427	−2.843
	Immediate	3-months follow-up	0.059	0.95	−1.230	1.348
		6-months follow-up	−0.506	0.25	−1.799	0.786
	3-months follow-up	6-months follow-up	−0.565	0.74	−1.857	0.727

^a^Tukey’s HSD Post hoc test. The mean difference is significant at the 0.05 level

### Primary outcome

**Fig. 4:**
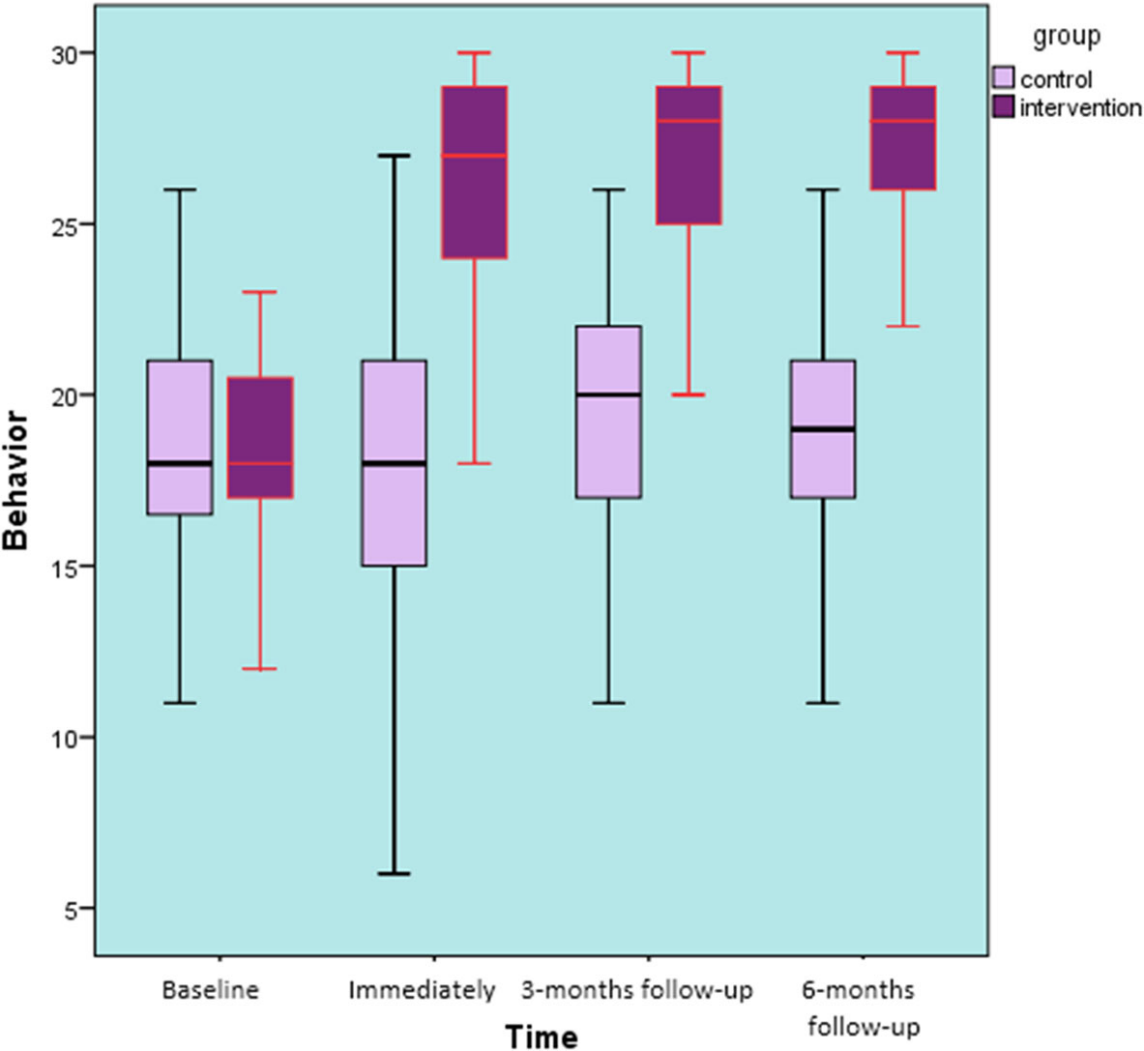
Back care behavior over 6 months

Back care-related behavior: At baseline, there was no significant difference in back-related behavior between the intervention group and the control group (t = 0.925, *P* = 0.36). There were significant differences between the study groups and over time (F ^(3, 291)^ = 29.266, *p* < 0.001) with a large effect size (η_p_^2^ = 0.22). Pupils in the intervention group scored higher on self-reported back-care behavior than controls at the three follow-up points (Fig. 4). Overall there was a 32% improvement in back-care behavior from baseline to immediate post-intervention and follow-up assessments. There was no significant difference in the back-related behavior scores among the control group from baseline to immediate post-intervention, 3-months and 6-months follow-ups (*F* = 0.496, *p* = 0.68).

### Secondary outcomes

**Fig. 5:**
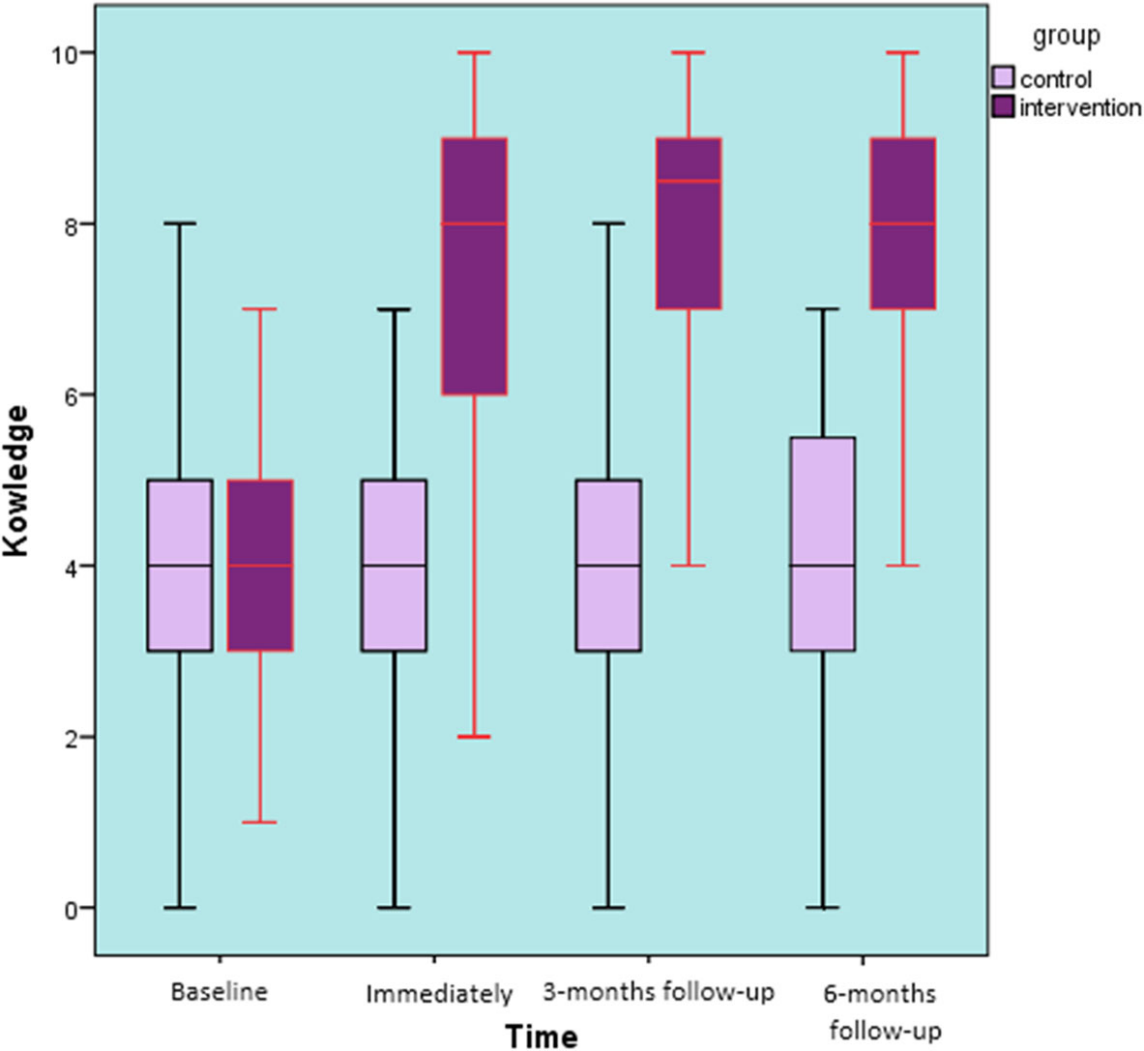
Back care knowledge over 6 months

Knowledge: Comparing the baseline back care knowledge, there were no significant differences between the study groups (t = 0.461, *p* = 0.65). The results revealed a significant interaction between the factors ‘group’ and ‘time’ of testing (F ^(3, 291)^ = 29.39, *p* < 0.001, η_p_^2^ = 0.21). The intervention group had an improvement on back care knowledge at the three follow-up assessments (overall increased by 36.4%) (Fig. 5). The only question that showed no significant difference from baseline to immediate post-intervention and follow-ups in the intervention group was question 3, ‘Which is the best way to carry your book bag?’ (*p* = 0.71). Likewise, there was no significant difference in the back-care knowledge mean scores of the control group from the baseline to immediate post-intervention, 3-months and 6-months follow-ups (*F* = 0.264, *p* = 0.85).

**Fig. 6:**
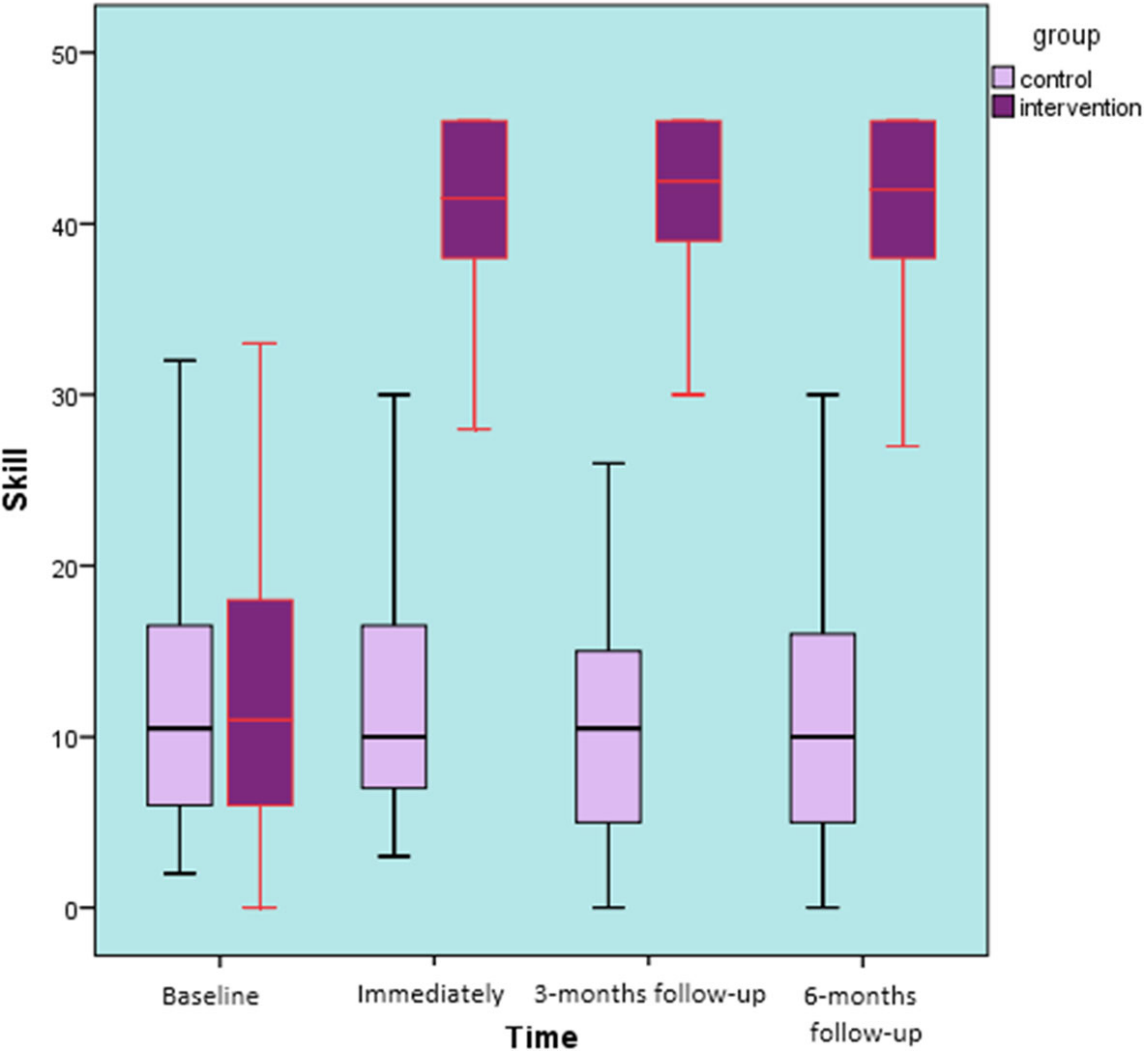
Back care skills over 6 months

Skills: Comparing the baseline back care skills, there were no significant differences between the study groups (t = 0.061, *p* = 0.95). Children in the intervention group demonstrated better skills than comparison pupils at 6-months follow-up (Fig. 6). More specifically after the back care programme the intervention group showed an improvement by 53.2% was observed. The interaction effects (time × group) on skills showed a significant interaction between the factors ‘group’ and ‘time’ (F ^(3, 291)^ = 32.04, *p* < 0.001, η_p_^2^ = 0.25).

**Fig. 7:**
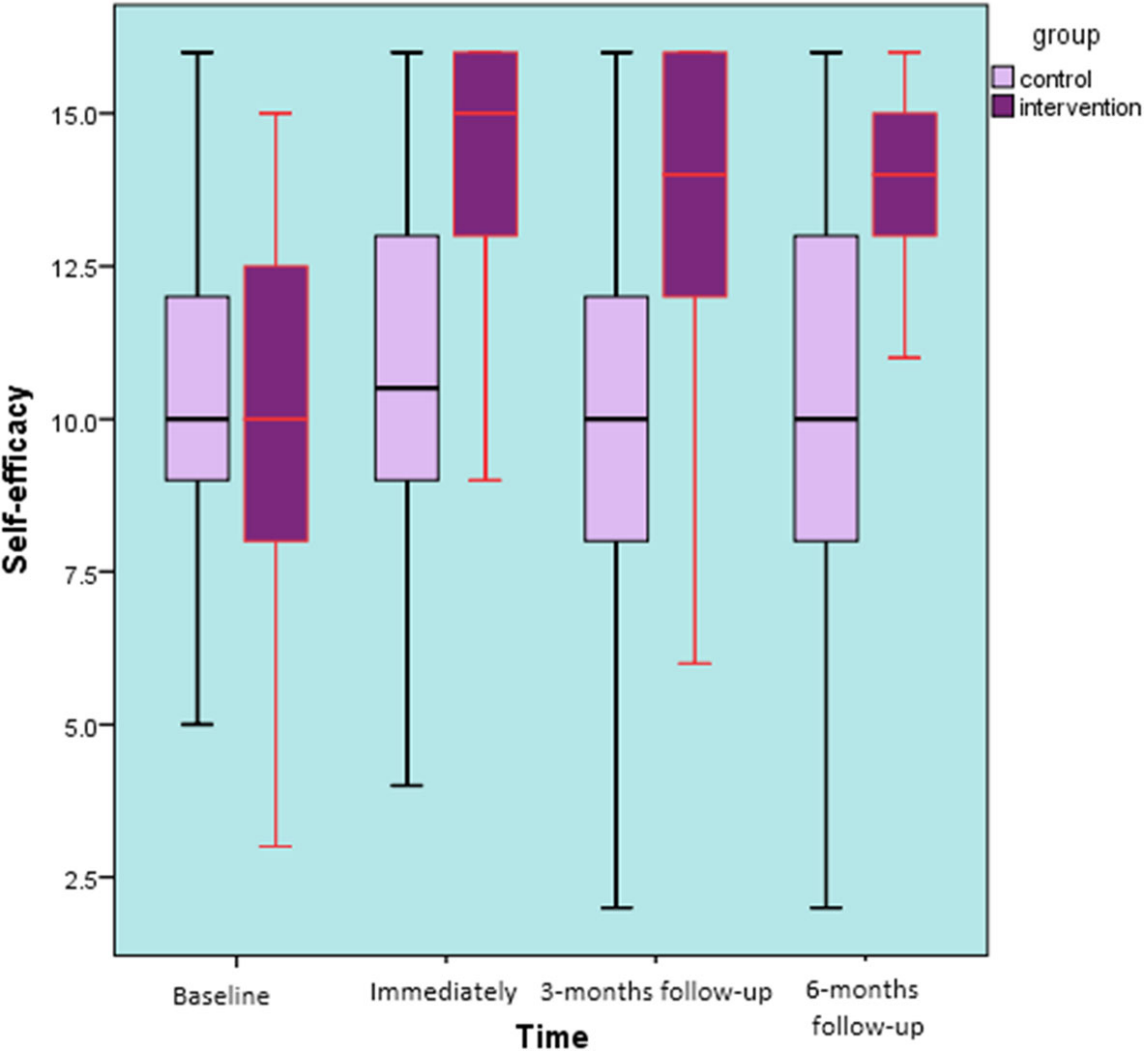
Back care self-efficacy over 6 months

Self-efficacy: At baseline, there was no significant difference in self-efficacy between the intervention group and the control group (t = − 0.441, *P* = 0.66). SE was improved by 19% from the baseline to follow-up assessments in the intervention group (Fig. 7). There were significant differences between the study groups and over time (F ^(3, 279)^ = 9.99, *p* < 0.001) with a medium effect size (η_p_^2^ = 0.11). The Tukey’s HSD post hoc test showed that pupils of the intervention group who answered the questionnaires at the immediate post-intervention, 3-months, and 6-months follow-ups, had significant higher mean scores of self-efficacy compared to the baseline (Table 4). By contrast, participants of the control group didn’t have significant higher mean scores of self-efficacy at the immediate post-intervention, 3-months, and 6-months follow-ups compared to the baseline (*F* = 0.788, *p* = 0.50).

**Fig. 8:**
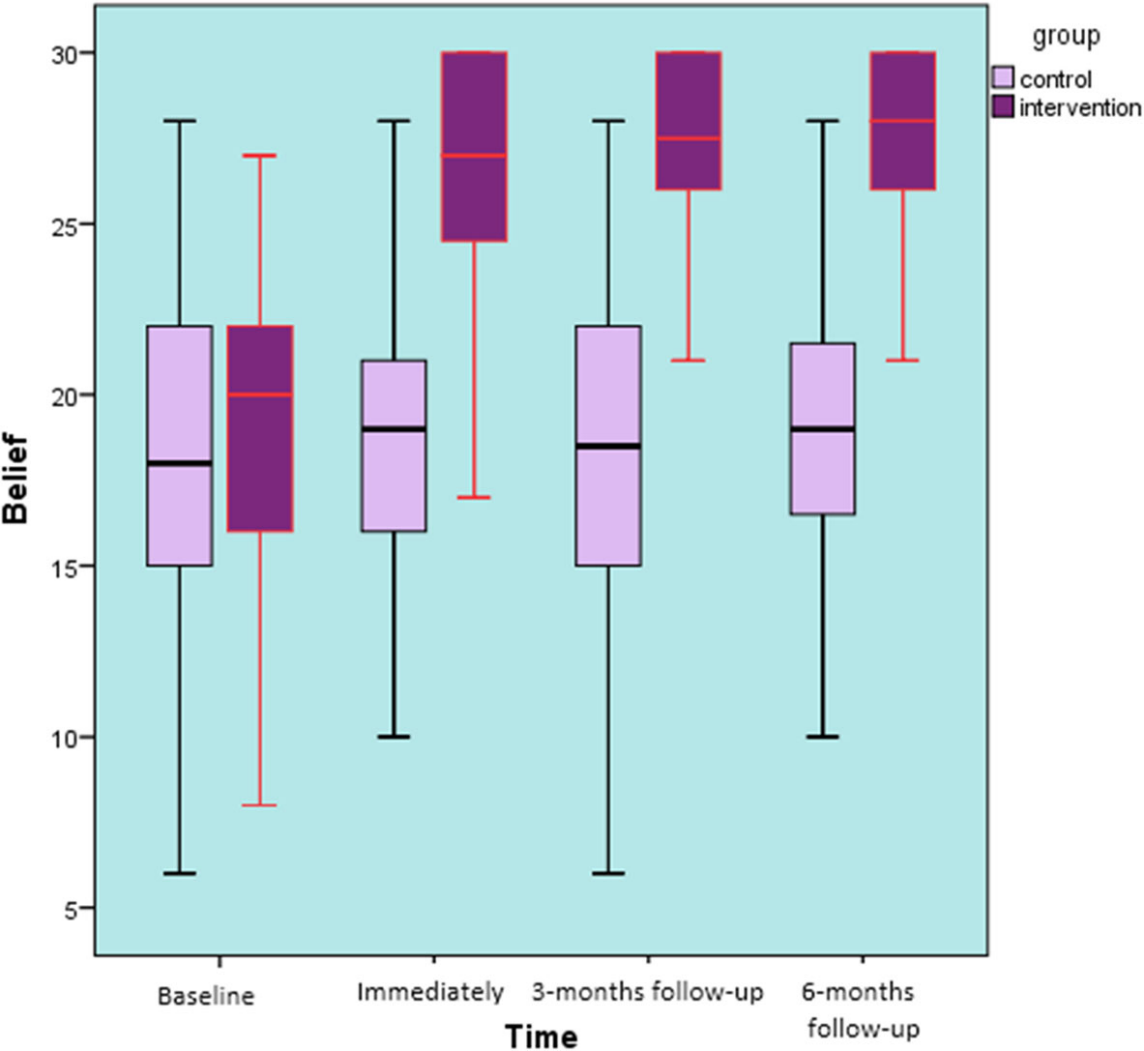
Beliefs over 6 months

Beliefs: Comparing the baseline beliefs, there were no significant differences between the study groups (t = − 1.193, *p* = 0.24). Finding revealed a significant interaction effect between ‘group’ and ‘time’ (F ^(3, 258)^ = 14.692, *p* < 0.001, η_p_^2^ = 0.14). Analysis of scores demonstrated that the educational program improved the beliefs in the intervention group (an increase of 25.6%) from baseline, to the 6-months follow-up (Fig. 8).

## Discussion

The main purpose of this study was to explore the effectiveness of the T-Bak educational program on back-related behavior among pupils. The trial showed that offering a SCT-based educational program could have a positive effect on primary and secondary outcomes in the intervention group compared with that in the control group. From the baseline to the six-month follow-up, the participants who also received the T-Bak educational program scored significantly higher than the controls with regard to the outcomes.

The present study demonstrated that the intervention group improved their healthy back behavior (32%) compared with the control group (with a large effect size = 0.22). Majority of those in the intervention group (51.9%) also reported that they had checked the weight of their schoolbags frequently during the 6-month follow-up evaluation. Likewise, Cardon et al. [12] had further argued more reports regarding book bag weight checking in the intervention group. Rodríguez-oviedo et al. had similarly developed a multi-faceted intervention for Spanish schoolchildren aimed to reduce the weight of their backpacks. They had found that 22.8% of the participants in the intervention group were not carrying a backpack exceeding 10% of their body weight [7]. In addition, 80.8% of those in the intervention group had reported that they were carrying an object with the load close to the body frequently during 3 and 6-month follow-up evaluations. In line with the present study, Geldhof et al. had found that 77% of individuals in the intervention group had reported that they were carrying an object as close as possible to their body during a 2-year follow-up [26].

Consistent with previous studies [12, 13, 17], the intervention group performed better on back care-related knowledge compared with the control group and also retained it over 6 months. Back care-related knowledge in the intervention group additionally improved by 36.4%, while the control group demonstrated no enhancements indicating that the findings were more promising than those in previous studies [12, 17]. The findings of the T-Bak educational program implied that the intervention group did not perform significantly better to carry book bag (over two shoulders) as compared with the control group due to the fact that most participants knew that they needed to use both straps (between 88.5 to 94.2% over 6 months in both groups); therefore, there was no significant difference between the study groups. These results were in agreement with previous studies [7, 12, 17].

In a study by Santos et al., assessing short- and medium-term effects of a posture educational program for pupils regarding knowledge and performance, improvements were found in post-test and follow-up assessments of the intervention group although they were not significant [21]. Similarly, Dullien et al. demonstrated that back care-related knowledge and parts of behavior had only enhanced in the intervention group from pre- to post-test stages [3].

The present study indicated that self-efficacy towards healthy back behavior had boosted in the intervention group (19.5%) as compared with the control group (with a medium effect size = 0.11). A possible explanation could be that most pupils in the intervention group perceived back care-related behavior as an easy issue at the baseline. Contrary to these findings, Dolphens et al. showed that an educational program had not changed behavior or self-efficacy while back care-related intervention had resulted in increased knowledge [13]. It should be noted that self-efficacy is assumed as one of the most important cognitive determinants known to affect both initiation and continuation of a behavior [13]. This enhancement in self-efficacy may thus suggest that the T-Bak intervention used goal-setting, modeling, feedback, and verbal persuasion adequately, since these strategies are important to improve self-efficacy in health-related behavior. It may be also due to strong self-judgment of back-related behavior. However, this improvement in self-efficacy denoted that the present findings were better than previous ones [12, 13].

Likewise, the results revealed that the T-Bak educational program was useful for boosting skills and beliefs in the intervention group as expected, since they respectively improved by 53.2 and 25.6%, which were better than those in previous studies [12, 13]. Improvement of back care-related skills might be due to the use of practical stations and demonstration/re-demonstration methods as innovative approaches to focus on back care tasks. To perform a healthy back care-related behavior, pupils must accordingly know what to do and how to do it. Therefore, promotion of mastery learning through skills training causes children succeed in attainable but increasingly challenging performances of healthy back care-related behaviors. The experience of performance mastery is also the most important effect on perceived self-efficacy.

Improvement in beliefs was probably due to the active approach of focusing on pain and utilization of group discussion, role-play, and recording pupils’ thoughts and experiences about back pain. Therefore, knowledge about benefits of healthy back care-related behavior and back pain prevention leads to building correct beliefs. Children also learn how to adopt a healthy back care behavior benefiting them.

The present study had some limitations that must be noted. First, the study was limited to the main psychological determinants of behavior within the SCT and other constructs (namely, environmental determinants of behavior) were not almost considered at all. Another potential concern was the fact that the data were only collected from the population of the 5th-grade girls enrolled in public elementary schools; therefore, the generalizability of the outcomes to the overall population might be limited. In addition, self-reported back pain was limited to within the last week to decrease recall bias.

## Conclusion

The findings from this study demonstrated that the T-Bak educational program is effective in improving back care related behaviors and it worth to be considered for primary school pupils. Further work is required to examine environmental determinants for inclusion and improvement of the T-Bak innovation.

## Data Availability

The datasets used and analyzed during the current study are available from the corresponding author on reasonable request.

## Abbreviations

BB: Back care-related behavior
η_p_^2^: Partial Eta Squared
Q&A: Question and answer
RCT: Randomized controlled trial
SCT: Social cognitive theory
SD: Std. deviation
SE: Self-efficacy
