# Correction to: Effectiveness of a theory-based back care intervention on spine-related behavior among pupils: a school-based randomised controlled trial (T-Bak study)

**DOI:** 10.1186/s12889-020-09200-8

**Published:** 2020-08-11

**Authors:** Zahra Akbari-Chehrehbargh, Sedigheh Sadat Tavafian, Ali Montazeri

**Affiliations:** 1 Department of Health Education, Faculty of Medical Sciences, Tarbiat Modares University, Tehran, Iran; 2 Health Metrics Research Center, Iranian Institutes for Health Sciences Research, ACECR, Tehran, Iran; 3 Faculty of Humanity Sciences, University of Science & Culture, ACECR, Tehran, Iran

**Correction to: BMC Public Health 20, 805 (2020)**

**https://doi.org/10.1186/s12889-020-08566-z**

It was highlighted that in the PDF version of the original article [1] an equation was missing under the Sample size section of the Methods. This Correction article shows the correct Sample size section of the Methods. The original article has been updated.

**Sample size**

Sample size estimation was based on previous study on back care education [14]. Based on this study (pre SD = б_1_ = 4.82 & post SD = б_2_ = 4.66) and expecting at least 2-unit difference in mean score of pre and post back care related behavior in intervention group (μ_1_-μ_2_); the following formula was used to estimate sample size. 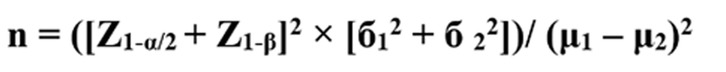


As such a study with 46 participants per group would have 80% power (β = 0.2) at 5% significance level (α = 0.05). However, allowing for a 10% dropout a sample of 52 pupils for each group was thought (in all 104).
